# Full Mass Range ΦSDM Orbitrap Mass Spectrometry for DIA Proteome Analysis

**DOI:** 10.1016/j.mcpro.2024.100713

**Published:** 2024-01-04

**Authors:** Sophia Steigerwald, Ankit Sinha, Kyle L. Fort, Wen-Feng Zeng, Lili Niu, Christoph Wichmann, Arne Kreutzmann, Daniel Mourad, Konstantin Aizikov, Dmitry Grinfeld, Alexander Makarov, Matthias Mann, Florian Meier

**Affiliations:** 1 Department Proteomics and Signal Transduction, Max Planck Institute of Biochemistry, Martinsried, Germany; 2 Thermo Fisher Scientific (GmbH), Bremen, Germany; 3 Department Clinical Proteomics, NNF Center for Protein Research, University of Copenhagen, Copenhagen, Denmark; 4 Department Computational Systems Biochemistry, Max Planck Institute of Biochemistry, Martinsried, Germany; 5 Functional Proteomics, Jena University Hospital, Jena, Germany

**Keywords:** Orbitrap, ΦSDM, proteomics, data-independent acquisition, high throughput

## Abstract

Optimizing data-independent acquisition methods for proteomics applications often requires balancing spectral resolution and acquisition speed. Here, we describe a real-time full mass range implementation of the phase-constrained spectrum deconvolution method (ΦSDM) for Orbitrap mass spectrometry that increases mass resolving power without increasing scan time. Comparing its performance to the standard enhanced Fourier transformation signal processing revealed that the increased resolving power of ΦSDM is beneficial in areas of high peptide density and comes with a greater ability to resolve low-abundance signals. In a standard 2 h analysis of a 200 ng HeLa digest, this resulted in an increase of 16% in the number of quantified peptides. As the acquisition speed becomes even more important when using fast chromatographic gradients, we further applied ΦSDM methods to a range of shorter gradient lengths (21, 12, and 5 min). While ΦSDM improved identification rates and spectral quality in all tested gradients, it proved particularly advantageous for the 5 min gradient. Here, the number of identified protein groups and peptides increased by >15% in comparison to enhanced Fourier transformation processing. In conclusion, ΦSDM is an alternative signal processing algorithm for processing Orbitrap data that can improve spectral quality and benefit quantitative accuracy in typical proteomics experiments, especially when using short gradients.

## Highlights

• ΦSDM signal processing increases Orbitrap mass resolution (or speed) >2-fold.
• GPUs enable real-time ΦSDM processing of full mass range spectra.
• ΦSDM resolves interfering signals in complex DIA spectra.
• Increased identification rates in short gradients.

## Graphical Abstract

**Figure:**
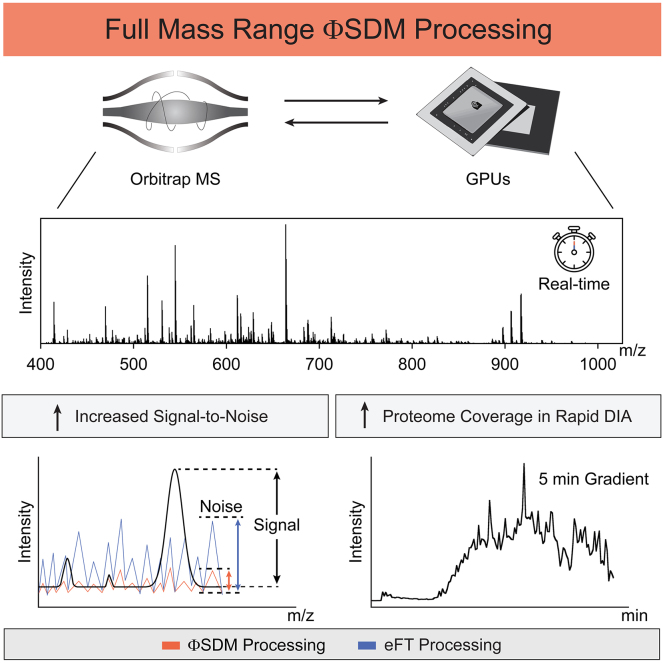

## In Brief

We describe the full mass range application of the ΦSDM signal processing algorithm for Orbitrap mass spectrometry in real time and evaluate its performance for label-free data-independent acquisition. ΦSDM increases the mass resolving power beyond the limits imposed by Fourier transformation, with advantages in areas of high spectral complexity and for fast chromatographic gradients. Our results suggest that it will be interesting to explore full mass range, real-time ΦSDM signal processing also for other applications of Orbitrap MS in proteomics research.

LC–MS has become the method of choice for the investigation of protein sequences and complex proteomes (1, 2). One of the most widely used mass analyzers for MS-based proteomics is the Orbitrap analyzer, first described in 2000 (3, 4, 5). In Orbitrap MS, the image current of trapped ions is recorded (“transient”) and converted into a high-resolution accurate mass spectrum using Fourier transformation (FT). As with other FT mass spectrometry (MS) analyzers, mass resolution scales with the transient duration, and even though enhanced FT (eFT) calculations enabled a twofold increase in mass resolving power using the same transient (6, 7), the mass resolution is inherently limited by the Fourier uncertainty. Interpolation techniques have been proposed to address this limitation; however, they lack the power to increase the spectral information content (8, 9). Only more recently, several approaches in ion cyclotron resonance MS have succeeded and are able to provide the required mass resolution at shorter transients (10, 11, 12, 13, 14). In particular, a novel computational strategy for processing Orbitrap transients, termed phase-constrained spectrum deconvolution method (ΦSDM), has the potential to double the mass resolving power at a given Orbitrap transient and could thereby significantly improve spectral quality and acquisition speed (15, 16). ΦSDM has already been implemented in the acquisition software of the most recent Orbitrap mass spectrometers (17, 18); however, because of the computational cost associated with the processing algorithm, its application has so far been limited to a narrow *m/z* region, such as the *m/z* range of tandem mass tag reporter ions (19, 20).

Here, we reasoned that a full mass range implementation of ΦSDM should be highly beneficial for data-independent acquisition (DIA), which has become a key driver of advancements in MS-based proteomics in recent years (21, 22). First popularized on a quadrupole time-of-flight instrument (21), DIA strategies have now been established on a multitude of mass analyzers (23, 24, 25, 26, 27, 28, 29). Unlike data-dependent acquisition (DDA), DIA does not sequentially fragment the top N most abundant peaks but cycles through the entire *m/z* range using isolation windows of defined width to simultaneously fragment all detectable precursors in each window. However, optimizing DIA methods often requires a compromise between spectral complexity and cycle time associated with a tradeoff between proteome coverage and quantitative accuracy (22, 24). In Orbitrap MS, narrow isolation windows and high mass resolution reduce complexity, improving spectral deconvolution, but this comes at the cost of longer cycle times and therefore a decrease in the ability to accurately quantify chromatographic peaks. To address this, here we investigated the potential of full mass range ΦSDM for DIA proteomics. In particular, we tested the compatibility with high-throughput DIA MS strategies using short LC gradients.

## Experimental procedures

### Sample Preparation

Human cervix carcinoma (HeLa) cells were cultured in Dulbecco’s modified Eagle's medium (Life Technologies Ltd) containing 20 mM glutamine, 10% fetal bovine serum, and 1% penicillin–streptomycin. After harvest, the cells were resuspended in PreOmics lysis buffer and incubated at 95 °C for 10 min to reduce disulfide bridges, alkylate cysteine residues, and denature proteins. Samples were sonicated using a rod sonicator (Branson SFX 250 Digital Sonifier) and subsequently incubated at 95 °C for an additional 5 min. HeLa cell lysates were diluted with an equal volume of water and digested overnight using equal amounts of LysC and trypsin (1:100 ratio at protein level). Following digestion, peptides were acidified to a final concentration of 1% TFA and purified on StrataTM-X-C (Polymeric Strong Cation) cartridges. Peptides were eluted in 80% acetonitrile (ACN)/1.25% NH_4_OH and subsequently dried using a SpeedVac (Eppendorf). Samples were resuspended in buffer A∗ (0.1% TFA, 2% ACN, or buffer A [0.1% formic acid (FA)]), for measurement with the Thermo Scientific EASY-nLC 1200 system or the Evosep LC system, respectively. Peptide concentrations were estimated by measuring absorbance at 280 nm on a Thermo Scientific NanoDrop 2000 spectrophotometer. For online MS injection using the Evosep One (LC) system, peptides were loaded onto Evotips according to the manufacturer’s instructions.

### High-pH Reverse-Phase Fractionation for Spectral Library Generation

For the short-gradient DIA experiments, gradient-specific spectral libraries were generated from 48 high-pH reverse-phase fractions for each gradient (5, 12, and 21 min) using a “spider” low-flow fractionator (30). The fractions were dried using a SpeedVac and resuspended in buffer A for Evotip loading and subsequent LC–MS analysis using the Evosep One system. We chose the peptide input amount for fractionation based on the injection amounts used for each gradient length. Peptide concentrations were estimated using a NanoDrop 2000 spectrophotometer, and 200, 100, and 50 ng per fraction were loaded on Evotips for the 60 samples per day (SPD), 100 SPD, and 200 SPD LC methods.

### LC–MS

All data were acquired on a Thermo Scientific Orbitrap Exploris 480 mass spectrometer (17). Standard LC measurements were performed using a Thermo Scientific EASY-nLC 1200 system, and an Evosep LC system (31) was used for preprogrammed short gradients with gradient lengths of 21, 12, and 5 min (60, 100, and 200 SPD). For the EASY-nLC chromatography system, we used an in-house packed 50 cm, 75 μm i.d. capillary column with 1.9 μm Reprosil-Pur C18 beads (Dr Maisch) and a laser-pulled electrospray emitter. The column temperature was maintained at 60 °C (sonation column oven). For the 120 min nLC gradient, mobile phase A was water with 0.1% FA, and mobile phase B was 80% ACN and 0.1% FA in water. Peptides were separated at a constant flow rate of 300 nl/min with a linear gradient of 5 to 30% mobile phase B within 95 min, followed first by a linear increase from 30 to 65% mobile phase B within 5 min and then a linear increase from 65 to 95% within another 5 min, where it was kept for 5 min before re-equilibration. Evosep measurements for 60 and 100 SPD (preprogrammed gradients) were performed using an in-house packed 8 cm, 150 μm i.d. capillary column with 1.9 μm Reprosil-Pur C18 beads (Dr Maisch). Column temperature was maintained at 20 °C. For the 200 SPD method, a commercial Evosep capillary column (EV1107) of 4 cm, 150 μm i.d. with 1.9 μm Reprosil-Pur C18 beads (Dr Maisch) was connected to an Evosep 30 μm i.d. stainless steel emitter (EV1086). Column temperature was maintained at 40 °C using a butterfly oven (Phoenix S&T). For both LC setups (EASY-nLC and Evosep One LC), in-house packed columns were interfaced with the Thermo Scientific NanoSpray Flex Ion Source, whereas the commercial column and emitter setup (for Evosep 200 SPD) was interfaced with the Thermo Scientific EasySpray Ion Source. For all measurements, spray voltage was set to 2400 V, RF level was set to 40, and the heated capillary temperature was set to 275 °C.

For EASY-nLC DIA, Orbitrap full MS scans were acquired from 400 to 1000 *m/z* at a resolution of 60,000 at *m/z* 200 with a normalized automated gain control (AGC) target of 200% and a maximum ion injection time of 45 ms. For MS/MS scans, the collision energy was set to 30%, the resolution to 15,000 at *m/z* 200, the normalized AGC target to 3000%, whereas the maximum injection time was set to “auto,” and the mass range was *m/z* 400 to 1000. For a theoretical cycle time of 3 s, 82 DIA windows of 7.3 *m/z* and an overlap of 1 *m/z* were used. For Evosep One LC DIA measurements, we designed gradient-specific methods. The general method settings for full MS and MS/MS were as aforementioned, except for the full MS AGC target, which was set to 300%. Cycle times and window placement were optimized according to the expected peak width (as reported by Spectronaut (Biognosys) based on 1.7 ∗ full width at half maximum) of the different gradient lengths at 21, 12, and 5 min for 60, 100, and 200 SPD, respectively. For the 60 SPD method, 53 DIA windows of 11.3 *m/z* with an overlap of 1 *m/z* were used (∼2 s cycle time). For the 100 and 200 SPD methods, 38 DIA windows of 15.4 *m/z* with an overlap of 1 *m/z* were chosen (∼1.5 s cycle time). Experiments to generate Evosep gradient-specific spectral libraries were performed using a DDA top12 method. Full MS scans were acquired from 400 to 1000 *m/z* at a resolution of 60,000 at *m/z* 200 with a normalized AGC target of 300% and a maximum injection time of 25 ms. Precursor ions were isolated in a 1.3 Thomson window, normalized AGC target was set to 200% with a maximum injection time of 22 ms, and the normalized collision energy was set to 30%. Precursors with charge states of 1+ or above 5+ were excluded from sequencing, and the exclusion time for previously targeted precursors was set to 30 s. All Orbitrap mass spectra were recorded in centroid mode.

### Real-Time and Full Mass Range ΦSDM Signal Processing

The ΦSDM has previously been described and applied successfully to small *m/z* areas for improved mass resolution of tandem mass tag reporter ions (15, 19). In brief, the algorithm is capable of resolving spectral features beyond the limitation imposed by the Fourier uncertainty by deconvolving an observed standard eFT spectrum on a multiply refined frequency grid with the sinc function as its basis functions. The sinc function reflects the finite length of a transient signal and is completely characterized by its length (*i.e.*, known *a priori*). The ΦSDM spectrum is a solution that minimizes discrepancy between the model and the observed signals in sense of L2 norm, being subject to a phase constraint in a narrow interval around the precalibrated phase. To avoid overdetermination, the phase constraint is relaxed to form a cone. For the full mass range implementation of ΦSDM, we interfaced the instrument internal PC with additional graphics processing units (GPUs). ΦSDM settings were accessed through a research prototype Tune, version (3.1.279.9, Thermo Fisher Scientific). Before measurements, ΦSDM phase and noise levels were calibrated. ΦSDM processing was performed on the external GPUs (“on box”), the number of iterations was limited to 150, the noise threshold was set to 1.41, and version 2 of the backfilling approaches was applied.

### Raw Data processing

DDA raw files for the spectral library were analyzed, and the libraries were generated using the Pulsar algorithm in Spectronaut, version 15.6 with default settings. The 5 min library consisted of 26,822 precursor and 4196 protein groups, the 12 min library of 61,111 precursor and 6824 protein groups, and the 21 min library of 92,865 precursor and 8173 protein groups. Targeted data extraction from DIA raw files was performed with Spectronaut, version 15.6 (32). The “Protein LFQ Method” was set to MaxLFQ, “Data Filtering” to Q-value, the “Normalization Strategy” to local normalization, and “Row Selection” was based on Q-value percentile with a “Fraction” setting of 0.2. For library generation and direct-DIA analysis, raw files were searched against a target/decoy database of the human proteome (UniProt, September 2021) with and without isoforms (80,426 and 20,588 entries). Trypsin/P was selected to generate peptides, and a maximum number of two missed cleavages were allowed. For all searches, carbamidomethyl (C) was set as a fixed modification, and acetyl (protein N-term) and oxidations (M) were set as variable modifications. For the MS1 and MS2 mass tolerance, we used the default value for Orbitrap MS in Spectronaut (40 ppm). A 1% false discovery rate cutoff at precursor and protein levels was applied.

### Data analysis

Statistical analysis and data visualization of the Spectronaut output tables was performed in Python (version 3.8.8) using matplotlib, pandas, and seaborn. For the manual inspection of close proximity peptide signals, we used a custom Python script based on alpharaw to read RAW data, alphabase to process peptides and fragments, and alphaviz (33, 34) to visualize peptide to spectrum matches (https://github.com/MannLabs).

For the analysis of neighboring peaks, because the resolving power in Orbitrap MS is inversely proportional to √*m/z*, we first calculated a theoretical tolerance window as a function of *m/z* assuming a nominal resolution of 30,000 at *m/z* 200. The resolving power is calculated as *R* = (*m/z*)/(Δ*m/z*), with *m/z* being the *m/z* value of a given peak and Δ*m/z* being the smallest peak-to-peak distance still resolvable at a given resolving power. We used this tolerance window to select peaks in close proximity to all peaks in all MS2 spectra of a given LC–MS experiment. The neighboring peak pairs were then filtered for noise using 4% relative to the base peak as an abundance threshold and retaining only pairs for which one of the peaks was not greater than four times more abundant than the other one. The resulting peak neighbor pairs represent peak pairs that require a nominal resolving power of ≥30,000 to be resolved, and their *m/z* and interpeak distance can therefore be considered as a measure of resolving power (3, 35, 36, 37).

Signal-to-noise ratio (SNR) scatter plots were filtered for outliers with log2 SNRs of 13 and 14 or higher for the *x*- and *y*-axis, respectively. This was necessary because these outliers (supplemental Fig. S4*A*) represent instances, for which Spectronaut could not determine an empirical noise value for a given extracted ion chromatogram (XIC), resulting in an overestimation of the SNR.

### Experimental Design and Statistical Rationale

All experiments were performed using aliquots of the same HeLa digest to minimize confounders from preanalytical steps. The 2 h HeLa experiment for the analytical evaluation was performed in quadruplicates, whereas all short-gradient experiments were performed in triplicates. Evaluation of the effects of ΦSDM on spectral quality, however, was performed on a per-spectrum level over the averaged information of thousands of spectra in a single run. To benchmark the two alternative signal processing algorithms, we kept the MS method settings identical for each comparison, except for activating ΦSDM or not (eFT).

## Results

### Full Mass Range ΦSDM Computation

**Fig. 1:**
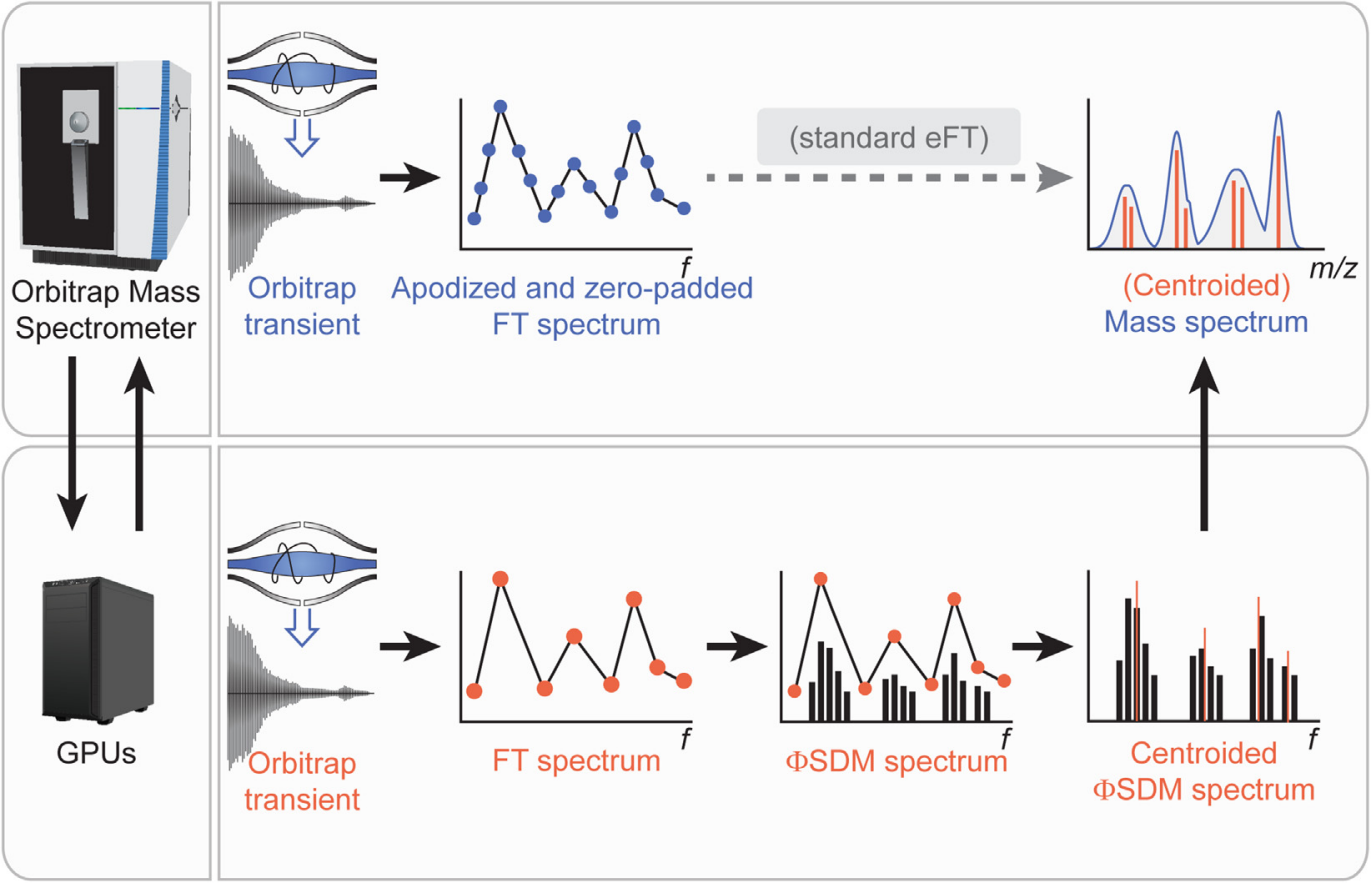
**ΦSDM for Orbitrap signal processing.** The image current induced on the detection plates of the Orbitrap by the oscillating ions is amplified and recorded as a transient signal followed by Fourier transformation (FT). With the assistance of an array of GPU cards to compensate for added computation costs, the resolution of the FT frequency spectrum is further enhanced by processing it with the ΦSDM. The ΦSDM spectrum is centroided, converted to the mass spectrum, and then stored in RAW format on the MS internal computer. ΦSDM, phase-constrained spectrum deconvolution method; GPU, graphics processing unit.

The ΦSDM can resolve signals in the mass spectrum that are closer than the limitation imposed by the Fourier uncertainty. This is achieved by iteratively fitting the observed signal to a refined frequency grid (15). To enable this computationally expensive method for the full mass range, we interfaced an Orbitrap mass spectrometer with GPUs for highly parallelized processing (Fig. 1). In our setup, the image current induced on the outer electrode of the Orbitrap analyzer (transient) is marshaled from the instrument’s internal computer to the GPUs. We reasoned that four Titan Xp Nvidia graphic cards installed on an auxiliary computer should provide sufficient resources to process multiple signals in parallel with an optimized CUDA C++ implementation of the ΦSDM algorithm. The calculated frequency spectrum is centroided and marshaled back to the instrument computer, where it is converted into a mass spectrum (4) and stored in the proprietary Thermo Fisher RAW file data format.

The key feature of ΦSDM is that it uses the phase as a constraint for signal deconvolution. To speed up the computation, making use of the very high stability of the MS electronics, we precalibrated the phase function externally as part of our weekly instrument maintenance routine. Furthermore, based on preliminary experiments, we parametrized the ΦSDM algorithm as detailed in the Experimental Procedures section and set the number of iterations to 150, which yielded a good compromise between processing speed and resolving power.

### Resolving Power of Full Mass-Range ΦSDM

**Fig. 2:**
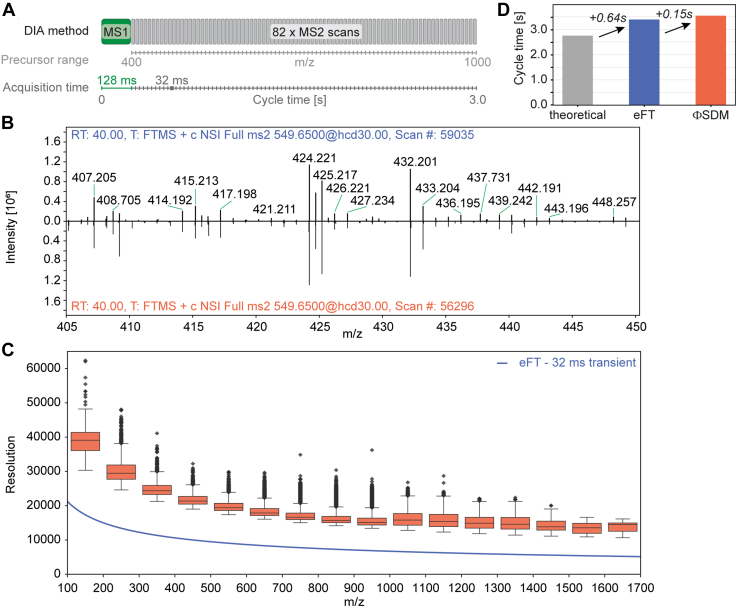
**Spectrum quality with ΦSDM in complex proteomics samples.** Analytical evaluation of ΦSDM and eFT signal processing using quadruplicate injections of a HeLa full proteome digest with a 2 h EASY-nLC gradient. *A*, data-independent acquisition (DIA) schema used to acquire both standard eFT and ΦSDM data. *B*, spectrum comparison for a representative eFT (*top*) and ΦSDM (*bottom*) DIA MS2 scan at a matching retention time and DIA isolation window. For inspection of areas of lower abundance ions, the *m/z* region 405 to 450 is shown. Full range spectra are provided in supplemental Fig. S1. *C*, Box–Whisker plot showing pairwise resolution of neighboring peaks with ΦSDM as compared with the nominal eFT resolution for an Orbitrap transient of 32 ms (*solid line*). See text for more details. *D*, comparison of summed transient time (*gray*) to experiment DIA cycle times for eFT (*blue*) and ΦSDM (*orange*). ΦSDM, phase-constrained spectrum deconvolution method; DIA, data-independent acquisition; eFT, enhanced Fourier transformation; MS, mass sprectrometry.

Having established an experimental setup that should be capable of processing full mass range spectra with ΦSDM in real time, we first inspected the resulting mass spectra with complex proteomics samples. For this, we analyzed the HeLa cell line proteome with 2 h gradients with DIA using either ΦSDM or eFT signal processing (Fig. 2*A*). Our DIA method comprised 82 equidistant isolation windows from *m/z* 400 to 1000 resulting in a cycle time of ∼3 s with transient times of 128 and 32 ms for full MS and MS/MS scans. These correspond to a nominal eFT resolution of 60,000 and 15,000 at *m/z* 200. Figure 2*B* shows two representative mass spectra for eFT *(upper panel*) and ΦSDM (*lower panel*) with matching retention time and isolation window between the two raw files. As expected, both spectra appeared very similar (Fig. 2*B* and supplemental Fig. S1). Upon closer inspection, we observed additional peaks in the ΦSDM spectrum in close proximity to peaks that ΦSDM and eFT had in common. To investigate the nature of these signals systematically, we parsed all MS2 spectra from a full LC–MS experiment with ΦSDM to find all neighboring peak pairs. Here, we defined close neighbors as *m/z* peak pairs with a distance that requires a resolving power ≥30,000 at *m/z* 200 to be resolved (see the Experimental Procedures section). In total, we observed >100,000 such peak pairs across the active part of the LC gradient (between scan #12,500 and #148,000) covering an *m/z* range between 100 and 1700. For these, we then calculated the theoretical resolving power required to distinguish them in a mass spectrum at full width half maximum (Fig. 2*C* and supplemental Table S1). The pairwise peak resolution across the *m/z* range in bins of 100 *m/z* followed the expected inverse proportionality between resolving power and √*m/z*, while exceeding the nominal eFT resolution by more than twofold. To further illustrate this point, we selected multiple peak pairs in a small *m/z* window of *m/z* 984 to 992 in the ΦSDM MS/MS spectrum #35,938 at a retention time of 25.5 min (supplemental Fig. S2). With eFT processing at a 32 ms transient, the resolving power in this *m/z* range is ∼7000, which means that two signals of equal abundance need to be at least 0.15 *m/z* apart to be resolved by eFT. Strikingly, all but one peak pair in this part of the ΦSDM spectrum were closer than 0.07 *m/z*, which equates a resolving power >13,000 in this *m/z* range or >30,000 at *m/z* 200.

Next, we investigated whether ΦSDM signal processing introduces extra scan overhead times. Comparing the empirical average cycle times with either eFT or ΦSDM processing to the sum of all Orbitrap transient times revealed overhead times of 0.39 and 0.54 s per scan cycle (Fig. 2*D*). This means that, even at an MS/MS scan rate of about 30 Hz, the additional data transfer to and back from the auxiliary computer as well as the iterative signal deconvolution caused only a minimal increase in cycle time of 0.15 s per 83 spectra. The comparison to eFT processing suggests that most of the overhead time can be attributed to AGC prescan events and ion routing. The ΦSDM processing time is mainly determined by the number of iterations to minimize the difference between modeled and observed signal. In our default setting, we limited the number of iterations to 150. To refine this, we varied the number of iterations from 100 to 200 in steps of 25, using the same 2 h LC gradient (supplemental Fig. S3). We observed a nearly linear increase in cycle time of ∼0.03 s for every additional 25 iterations, from 0.08 s for 100 iterations, to 0.20 s for 200 iterations. As the difference in cycle time between the 100 iterations and 150 iterations is negligible on the chromatographic time scale, all remaining datasets used 150 iterations.

### SNR and Mass Accuracy of Full Mass Range ΦSDM

**Fig. 3:**
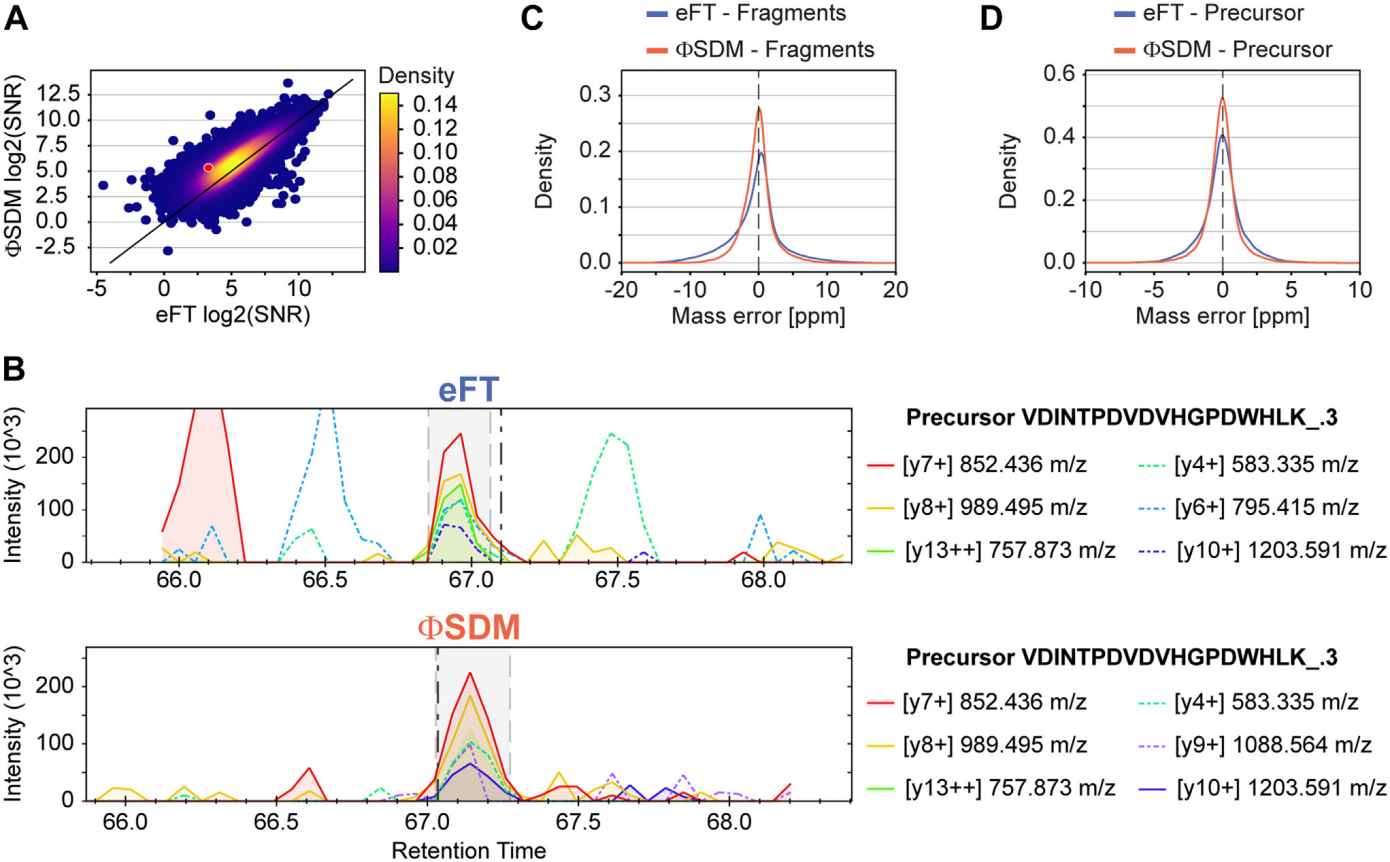
**Signal-to-noise (SNR) and mass accuracy in complex samples.***A*, scatter plot representing the log2 SNR comparison between eFT and ΦSDM. *Diagonal* indicated in *black* represents line of origin, and S/N distribution is colored based on density. Position of VDINTPDVDVHGPDWHLK_.3 peptide highlighted in *red*. *B*, comparison between extracted ion chromatograms (XICs) for precursor VDINTPDVDVHGPDWHLK_.3 from an eFT (*upper panel*) or ΦSDM (*lower panel*) run. *C*, comparison of calibrated mass error for all fragments identified in eFT (*blue*) and ΦSDM (*orange*). *D*, comparison of calibrated mass error for all precursors identified in eFT (*blue*) and ΦSDM (*orange*). ΦSDM, phase-constrained spectrum deconvolution method; eFT, enhanced Fourier transformation.

Having confirmed that ΦSDM achieves an at least twofold higher resolving power across the full mass range with minimal impact to the acquisition rate, we asked whether this benefits mass accuracy and SNR in a practical proteomics setting. We first analyzed the data with a 'directDIA' spectrum library and extracted SNRs. The Spectronaut software computes SNRs for identified peptides based on XICs, where signal is the maximum intensity of the summed fragment XICs within the chromatographic peak boundaries and noise is the average summed fragment XICs outside the peak boundaries. Figure 3*A* shows the logarithmized SNR for peptides shared between quadruplicate eFT and ΦSDM injections (see also the Experimental Procedures section). Our analysis revealed a substantial shift toward higher SNRs with ΦSDM (median ΦSDM to eFT ratio of 1.5, supplemental Fig. S4), suggesting that ΦSDM successfully resolves interfering signals from fragment ion traces (chemical noise). Figure 3*B* visualizes this effect for one example chosen from Figure 3*A* (*red dot*, ΦSDM:eFT ratio 2.0). The fragment XICs for the triply charged precursor ion of VDINTPDVDVHGPDWHLK showed low CVs in-between replicates and similar intensities in eFT (Fig. 3*B*, *upper panel*) and ΦSDM (Fig. 3*B*, *lower panel*), whereas the interfering signals were markedly reduced with ΦSDM in all four replicates (supplemental Fig. S5).

Next, we investigated the mass accuracy (after nonlinear recalibration) for ΦSDM in comparison to eFT both on the fragment (Fig. 3*C*) and precursor (Fig. 3*D*) ion level (supplemental Table S2). The mass error distribution was centered on 0 for both, and we observed only minor differences in shape and standard deviation between ΦSDM and eFT processing (supplemental Fig. S6). This confirms that ΦSDM signal processing does not affect mass accuracy, whereas the precision of mass spectral peak centroiding in proteomics practice appears primarily limited by the transient length rather than resolving power (38).

### Effect of ΦSDM on Identification Rates in Complex DIA Spectra

**Fig. 4:**
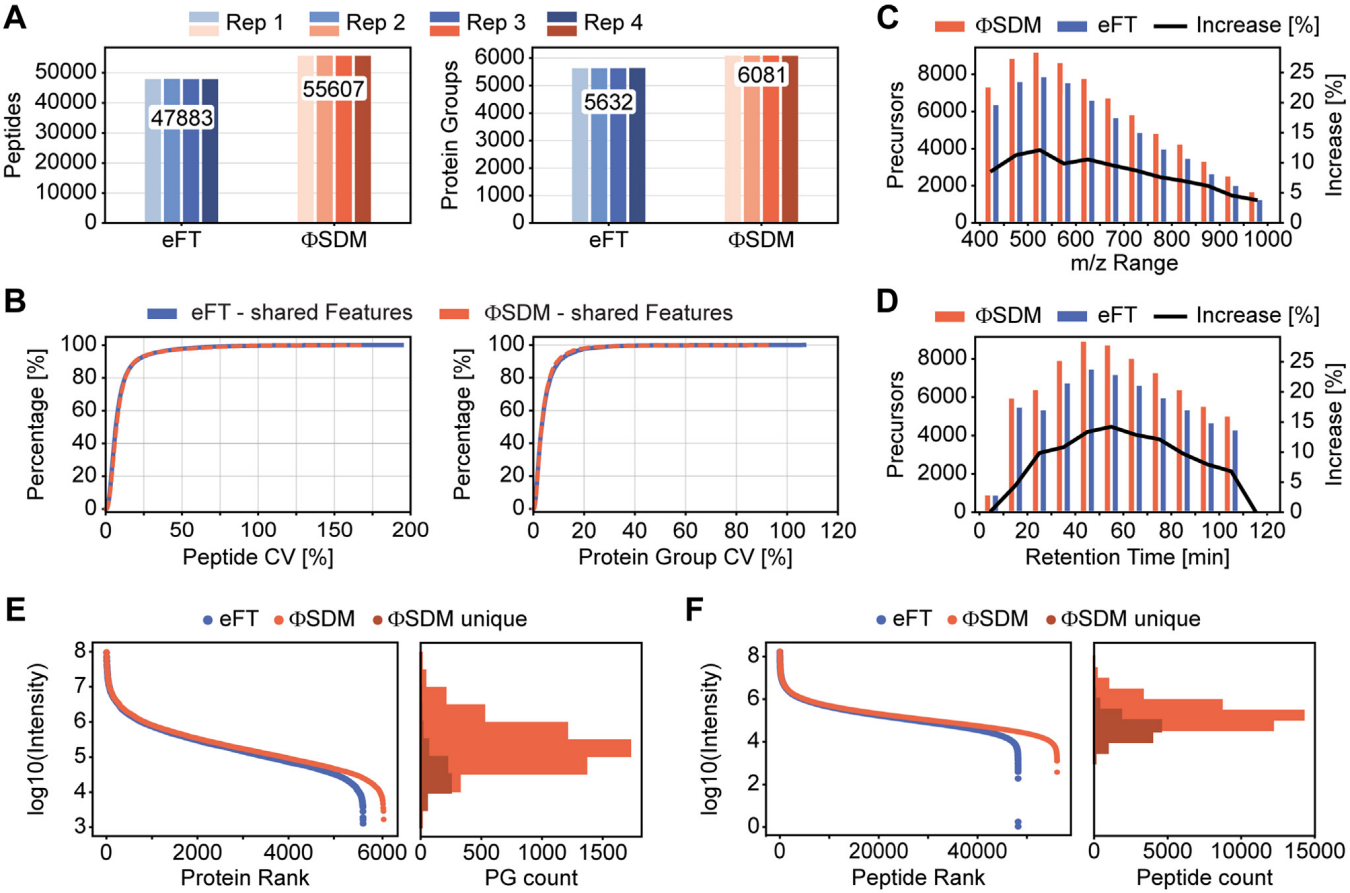
**Influence of ΦSDM on identification and quantification**. Analytical analysis of the influence of the ΦSDM and eFT processing method on spectral quality was performed using quadruplicate HeLa measurements on a 2 h nLC gradient. *A*, bar plots comparing the number of peptides (*left*) and protein groups (PGs; *right*) identified in quadruplicate measurements of 200 ng HeLa digest in the eFT (*blue*) and ΦSDM (*orange*) dataset. Mean number of identifications indicated. *B*, comparison of cumulative CV values for shared peptides (*left*) and PGs (right). CVs for peptides or PGs identified in eFT and ΦSDM are represented in *blue* and *orange*, respectively. *C*, bar chart of precursor identification for eFT (*blue*) and ΦSDM (*orange*) along the retention time dimension with a bin size of 10 min. Increase in identification (in percent [%]) for ΦSDM in comparison to standard eFT is indicated in *black*. *D*, bar chart of precursor identification for eFT (*blue*) and ΦSDM (*orange*) along the retention mass-to-charge (*m/z*) range with a bin size of 50 (*m/z*). Increase in identification (in percent [%]) for ΦSDM in comparison to standard eFT is indicated in *black*. *E*, abundance distribution (*left side*) of proteins identified in the eFT (*blue*) and ΦSDM (*orange*) datasets. Abundance is represented as the log10 scale median protein intensities. The slight shift toward lower abundance for proteins uniquely identified in the ΦSDM dataset (*red*) in comparison to those that are common between the ΦSDM and eFT datasets (*orange*) is highlighted in the histogram. *F*, abundance distribution (*left side*) of peptides identified in the eFT (*blue*) and ΦSDM (*orange*) datasets. The slight shift toward lower abundance for peptides uniquely identified in the ΦSDM dataset (*red*) in comparison to those that are common between the ΦSDM and eFT datasets (*orange*) is highlighted in the histogram. Abundance is represented as the log10 scale median peptide intensities. ΦSDM, phase-constrained spectrum deconvolution method; eFT, enhanced Fourier transformation.

Having established the analytical figures of merit, we investigated the influence of ΦSDM on peptide identification rates and label-free quantification accuracy in a typical DIA experiment (Fig. 4). In the quadruplicate 2 h HeLa experiments, on average, 47,883 and 55,607 peptides for eFT and ΦSDM were identified with 'directDIA' (Fig. 4*A*, *left panel*). This translated into an 8% improvement on the protein group level and over 6000 identified protein groups per replicate with ΦSDM (Fig. 4*A*, *right panel*). Irrespective of the signal processing method, we achieved an excellent quantitative reproducibility with median CV <8% on the peptide and <4% on the protein group level (Fig. 4*B*). Comparing only the subset of shared peptide identifications, we found similar median CVs of 7.1% and 6.6% for ΦSDM and eFT, respectively.

To delineate the higher identification rates with ΦSDM, we plotted the distribution of peptide ions in *m/z* and retention time. Figure 4*C* shows a consistent increase in the number of identified peptides throughout the binned precursor *m/z* range (bin size of 50 *m/z*). Interestingly, the largest relative increase of up to 12% was in the range of *m/z* 400 to 600, where most peptides were identified in absolute numbers. In contrast, in the higher *m/z* range with fewer peptides, the increase by ΦSDM was moderate. This result indicates that ΦSDM outperforms eFT particularly in areas of high peptide density. This is further supported by a comparison of identification rates along the retention time dimension (Fig. 4*D*). Again, the highest gains were in the center of the chromatographic gradient in RT bins with the overall highest number of identifications.

Peptide abundances with both eFT and ΦSDM spanned more than five orders of magnitude (Fig. 4*E*). Peptides uniquely identified in the ΦSDM experiments were distributed across the entire abundance range, even though a comparison with peptides that were in common between ΦSDM and eFT revealed a bias toward the mid-to-lower abundance range (histogram in Fig. 4*E*). Consequently, the protein groups uniquely identified in ΦSDM runs were distributed over the entire abundance range of about five orders of magnitude, but with a higher density in the lower abundance range (Fig. 4*F*). From this, we concluded that ΦSDM—while keeping all other experimental parameters constant—facilitates the detection of lower-abundance signals in complex samples such as full proteome digests.

### Rapid DIA Experiments With ΦSDM

**Fig. 5:**
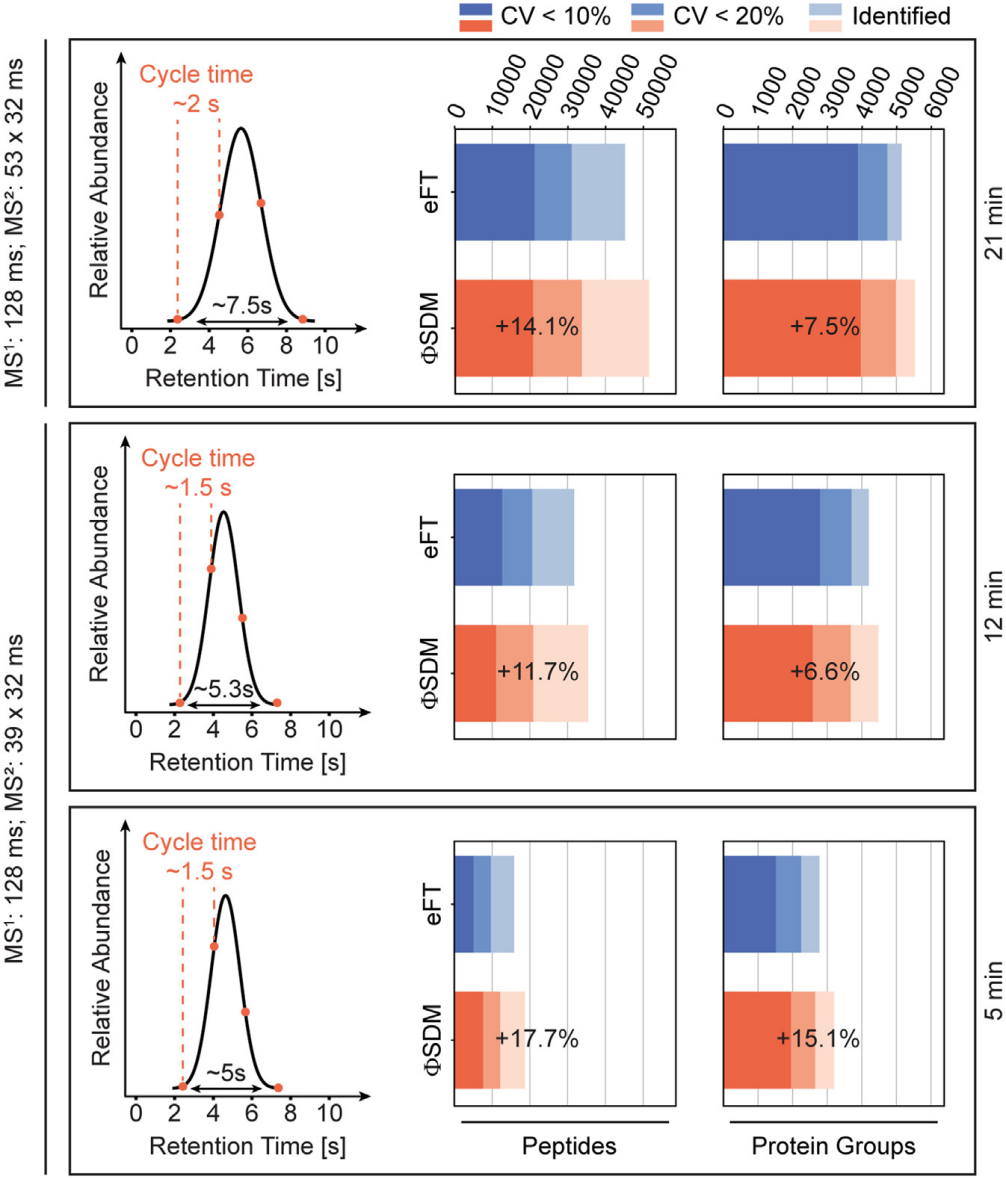
**ΦSDM for rapid DIA proteomics.** DIA acquisition schemas were optimized for each gradient to guarantee at least three datapoints per peak. Average peak width and chosen cycle time are shown in the *left panel*. Full MS, MS/MS transients, and number of DIA windows used to achieve the different cycle times (1.5 s for 200 and 100 SPD methods, 2 s for the 60 SPD method) are indicated on the *left*. Number of protein and peptide identification (*right panel*) in triplicates. HeLa measurements using the Evosep 5 min (200 SPD, *top*), 12 min (100 SPD, *middle*), and 21 min (60 SPD, *bottom*) gradient for eFT (*blue*) and ΦSDM (*orange*). Total identifications across the triplicates shown in *light blue* and *light orange* for eFT and ΦSDM, respectively. Proteins and peptides quantified with CV values <20% are shown in medium *blue/orange*, whereas those with CV values <10% are shown in *dark blue/orange* for eFT and ΦSDM, respectively. For the triplicate measurements, 100 ng HeLa were injected for the 5 min and 12 min gradients each, whereas 200 ng were used per injection for the 21 min gradient. ΦSDM, phase-constrained spectrum deconvolution method; DIA, data-independent acquisition; eFT, enhanced Fourier transformation; SPD, sample per day.

The field of MS-based proteomics is currently pushing for increasing throughput to facilitate large experimental designs and clinical studies (39, 40, 41, 42, 43). However, shortening LC gradients entails increased spectrum complexity as more peptides coelute and, in addition, accurate quantification of narrower chromatographic peaks requires fast detection systems. The most common strategies to accommodate this in (Orbitrap) DIA methods are to either decrease the number of DIA windows and thus increase the number of cofragmented peptides for a fixed total precursor mass range or to lower the mass resolution to achieve faster cycle times (22, 24). Our aforementioned results indicate that ΦSDM is most beneficial in dense regions of LC gradients. To test this hypothesis further, we turned to gradually shorter LC gradients compressing the peptide elution window. We used the Evosep One LC system to run preconfigured gradients for a throughput of 60, 100, and 200 SPD and designed DIA methods aiming for at least three data points per peak on average. The three gradients resulted on average in chromatographic peak widths of 5, 5.3, and 7.5 s (Fig. 5, *left column*). Accordingly, we adapted the number of DIA isolation windows in the *m/z* range 400 to 1000 to achieve cycle times around 1.5 s for the 200 and 100 SPD methods, and 2 s for the 60 SPD method, recording 128 and 32 ms transients for MS and MS/MS scans (supplemental Fig. S7*A*).

As our objective was to maximize the proteome coverage, we generated gradient-specific libraries with DDA from 48 high-pH reverse-phase fractionated HeLa samples per gradient. A database search using the Pulsar search engine integrated in the Spectronaut software resulted in 4196, 6824, and 8173 protein groups for the 200, 100, and 60 SDP gradients, respectively. Matching triplicate single-run measurements of 200 ng HeLa digest with both eFT and ΦSDM to the respective library, we observed an overall increase in peptide and protein group identifications by ΦSDM (Fig. 5). In line with our results for the 2 h gradient, we observed increasing SNRs even though this effect was attenuated for shorter gradients (supplemental Fig. S7*B*).

Consistently for all short gradients, ΦSDM increased the number of identified peptides over conventional eFT signal processing particularly in retention time and isolation bins with high peptide density (supplemental Fig. S8). From the 60 SPD gradient, we identified 45,201 peptides with eFT and 52,558 peptides with ΦSDM, from which 5151 and 5536 protein groups were inferred. Likely because of the still relatively long cycle time, the fraction of peptides and proteins quantified with a CV <10% remained constant, whereas we quantified slightly more proteins with a CV <20% in the ΦSDM experiment. Using the 100 SPD gradient and a DIA method with wider isolation windows resulted in 11.7% and 6.6% more peptide and protein group identifications with ΦSDM. In line with our starting hypothesis, we observed the highest benefits of ΦSDM for the 5 min gradient (200 SPD) with a 17.7% increase in peptide and 15.1% increase in protein group identifications. Here, we identified over 3000 protein groups (out of 4200 in the library) from triplicate injections of 100 ng, while maintaining a very good quantitative reproducibility with median CVs of 10% and 7% for peptides and protein groups.

## Discussion

The ΦSDM signal processing method for Orbitrap MS can achieve a more than twofold higher mass resolution than conventional eFT for the same transient length but was previously limited to narrow *m/z* ranges because of its high computational cost (19). Here, we have implemented ΦSDM on an auxiliary computer to parallelize data acquisition and signal processing in real time. This setup allowed us to extend ΦSDM to the full mass range with only minimal impact on the acquisition rate in DIA proteomics experiments and maintaining the high mass accuracy of the Orbitrap mass analyzer. Analyzing fragment ion peak pairs in complex spectra, we confirmed that ΦSDM increases the mass resolving power by more than twofold over conventional eFT in the full mass range. In DIA experiments of a human cancer cell lysate, this resulted in 50% increased SNRs, facilitating peptide identification and label-free quantification. Furthermore, we found increased identification rates in dense areas of chromatographic gradients, making the combination of DIA with ΦSDM particularly attractive for short LC gradients. While we here focused on increasing resolving power (keeping transient length constant), in such applications, it can be desirable to shorten the transient length (keeping resolving power approximately constant). The faster scan rate would then allow for more data points per peak (shorter cycle time) or lower spectral complexity by increasing the number of DIA windows per cycle.

Similarly, while we focused on label-free quantification in this study, we note that workflows using nonisobaric labeling or isobaric labeling with high-mass reporter ions should directly benefit from higher mass resolution (44, 45, 46, 47, 48). Moreover, faster scan rates open up opportunities for advanced DIA acquisition schemes that, for example, include BoxCar (49) scans for high dynamic range MS1 scans or cycle through multiple compensation voltages with field asymmetric ion mobility spectrometry (17, 50, 51, 52, 53). We also envision that ΦSDM could be even more beneficial for top–down proteomics as ion decay in the Orbitrap analyzer limits the practical maximum transient length. We thus conclude that full mass range and real-time ΦSDM signal processing is attractive for a wide range of MS-based proteomics applications.

## Data availability

The MS proteomics data have been deposited at the ProteomeXchange Consortium (http://proteomecentral.proteomexchange.org) *via* the PRIDE partner repository (54) and are available with the dataset identifier PXD044292.

## Supplemental data

This article contains supplemental data (Supplemental Figs. S1–S8; Supplemental Tables S1 and S2).

## Conflict of interest

K. L. F., A. K., D. M., K. A., D. G., and A. M. are employees of Thermo Fisher Scientific, the manufacturer of Orbitrap instrumentation used in this research. M. M. is an indirect investor in Evosep Biosystems. All other authors declare no competing interests.
