# Supplementary material for: Full Mass Range ΦSDM Orbitrap Mass Spectrometry for DIA Proteome Analysis
Source: Mol Cell Proteomics. 2024 Jan 4;23(2):100713. doi: 10.1016/j.mcpro.2024.100713 (PMC10851225; doi:10.1016/j.mcpro.2024.100713)
Supplement: Supplemental data [file mmc1.pdf]

Full Mass Range ΦSDM Orbitrap Mass Spectrometry for DIA Proteome Analysis

Sophia Steigerwald[1], Ankit Sinha[1], Kyle L. Fort[2], Wen-Feng Zeng[1], Lili Niu[3], Christoph Wichmann[4], Arne Kreutzmann[2], Daniel Mourad[2], Konstantin Aizikov[2], Dmitry Grinfeld[2], Alexander Makarov[2], Matthias Mann[1,3], Florian Meier[1,5]

[1]Department Proteomics and Signal Transduction, Max Planck Institute of Biochemistry, Martinsried, Germany

[2]Thermo Fisher Scientific (GmbH) Bremen, Germany

[3]NNF Center for Protein Research University of Copenhagen, Copenhagen, Denmark

[4]Department Computational Systems Biochemistry, Max Planck Institute of Biochemistry, Martinsried, Germany

[5]Functional Proteomics, Jena University Hospital, Jena, Germany

Content:

- Supplementary Figures 1-8
- Supplementary Tables 1-2

Supplementary Figures

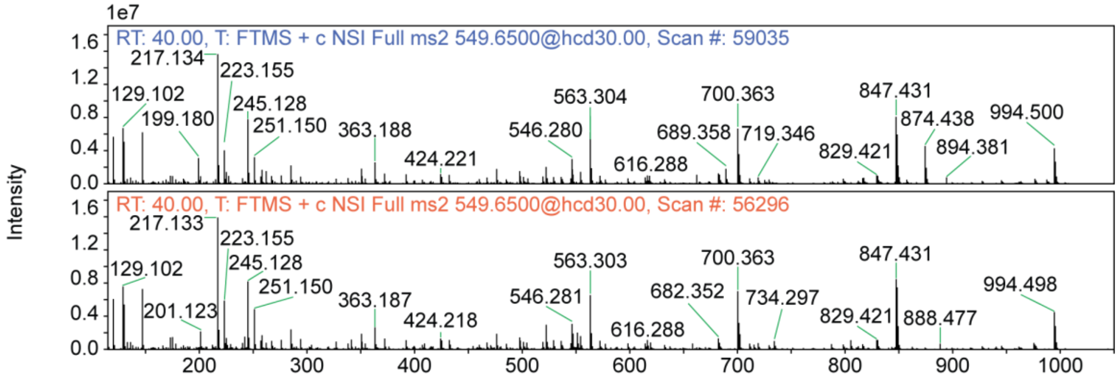

Supplementary Figure 1. Full range spectra for comparison of representative eFT (top) and ΦSDM (bottom) DIA MS2 scans at a matching retention time and DIA isolation window shown in Fig 2B.

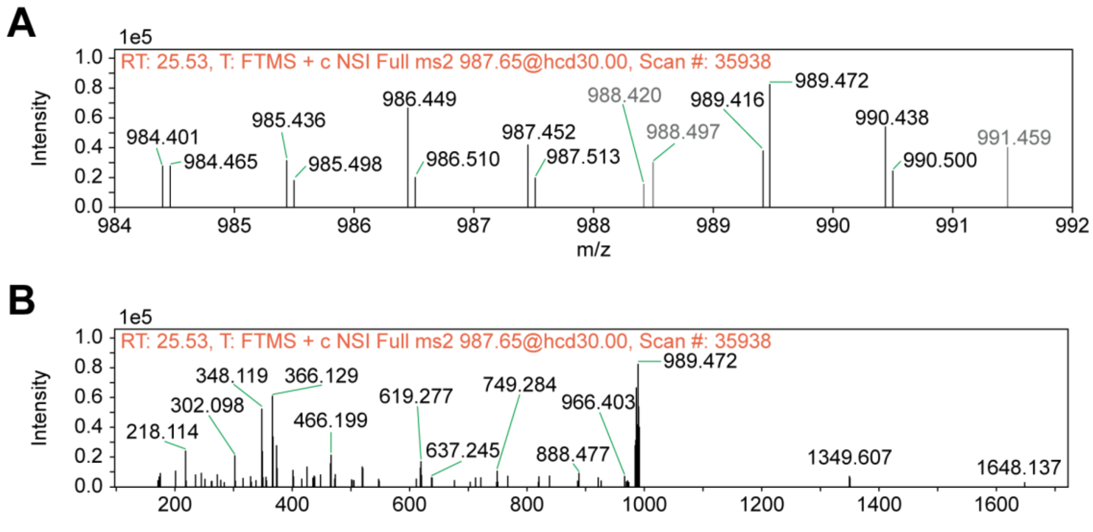

Supplementary Figure 2. Spectrum example for neighbor peak analysis in Fig. 2C. **(A)** Series of multiple peak neighbors in a small m/z window of m/z 984-992 in the ΦSDM MS/MS spectrum #35,938 at a retention time of 25.53 minutes. Grayed out peaks either don't have a peak neighbor or the peak pair require a resolving power of <13,000 in this m/z range or <30,000 at m/z 200 and is therefore not considered peak pairs. **(B)** Full range spectrum of A.

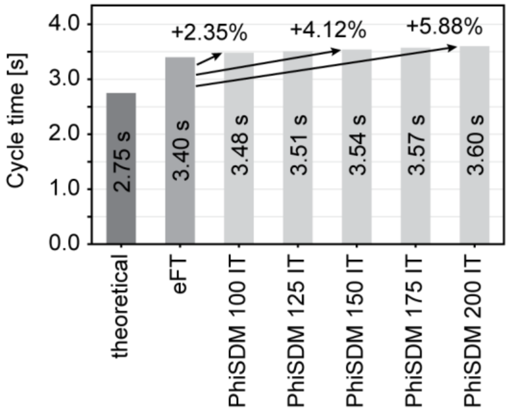

Supplementary Figure 3. Impact on the ΦSDM iteration setting on cycle time. Comparison of theoretical and experimental eFT and ΦSDM cycle times. ΦSDM processing time is primarily determined by the number of iterations allowed to minimize differences between modeled and observed signal. To evaluate the influence on this setting on experimental cycle time, we varied the number of iterations between 100 to 200 in steps of 25 (default setting: 150).

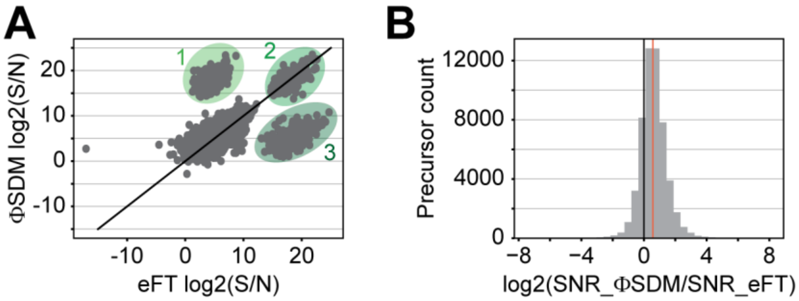

Supplementary Figure 4. Extended Signal-to-noise analysis on 2h gradients dataset. **(A)** Not-filtered scatter plot representing the log2 Signal to Noise (S/N) level comparison between eFT and ΦSDM. Diagonal indicated in black represents line of origin and precursor clouds highlighted in green represent different populations of software-based outliers. When Spectronaut cannot identify a noise value for a given XIC, a random value between 0 and 1 is used to simulate absence (no-value). Due to stringent intensity cutoffs in Orbitrap MS, the lowest possible noise value present in the Orbitrap MS data is much higher than one (true-value), resulting in exceptionally high SNRs in "no-value" situations. Precursor cloud one and three represent situations where no noise value could be identified for a given XIC in ΦSDM and eFT respectively (no-value vs. true-value), while precursor cloud two represents the situation where no noise value could be extracted from the XIC of both conditions (no-value vs. no-value). **(B)** Histogram depicting the precursor distribution across the log2 ratio of ΦSDM to eFT SNRs. Vertical zero-line depicted in black and median ΦSDM to eFT ratio indicated in red.

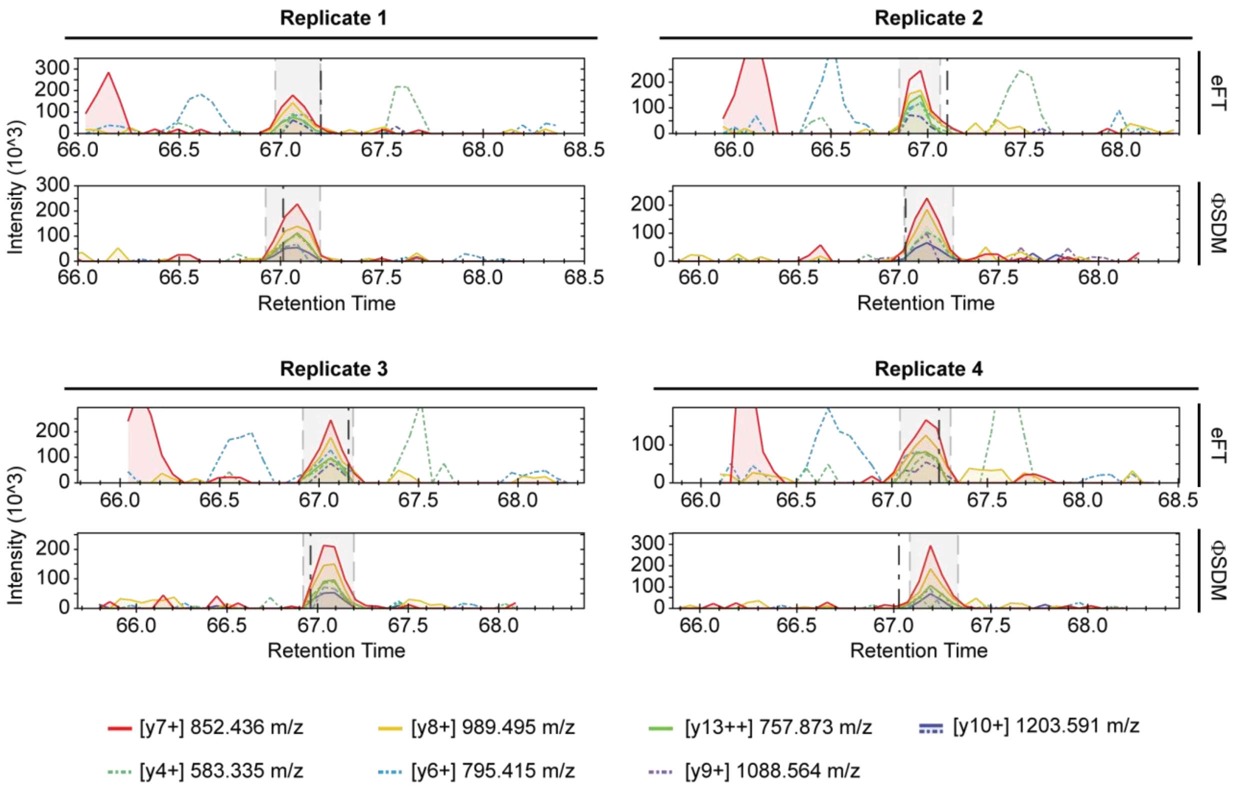

Supplementary Figure 5. Matched XIC comparison. Comparison between extracted ion chromatograms (XICs) for precursor VDINTPDVDVHGPDWHLK_.3 across all eFT or ΦSDM replicate runs. Replicates are matched between eFT (upper line) and ΦSDM (lower line). Dotted lines represent potential interference signals detected by Spectronaut, which are removed for relative quantification. The matched replicate two runs, were selected for demonstration in Figure 3B as they are optimally matched based on intensity levels.

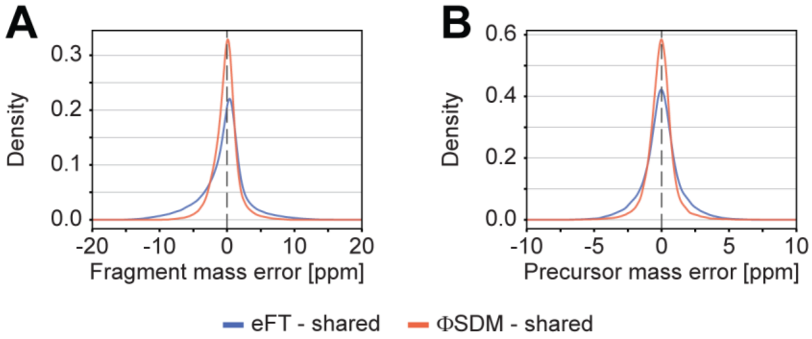

Supplementary Figure 6. Extended comparison of calibrated mass error between eFT and ΦSDM. **(A)** Comparison of calibrated mass error for fragments identified both in the eFT (blue) and ΦSDM (orange). **(B)** Comparison of calibrated mass error for precursors identified both in the eFT (blue) and ΦSDM (orange).

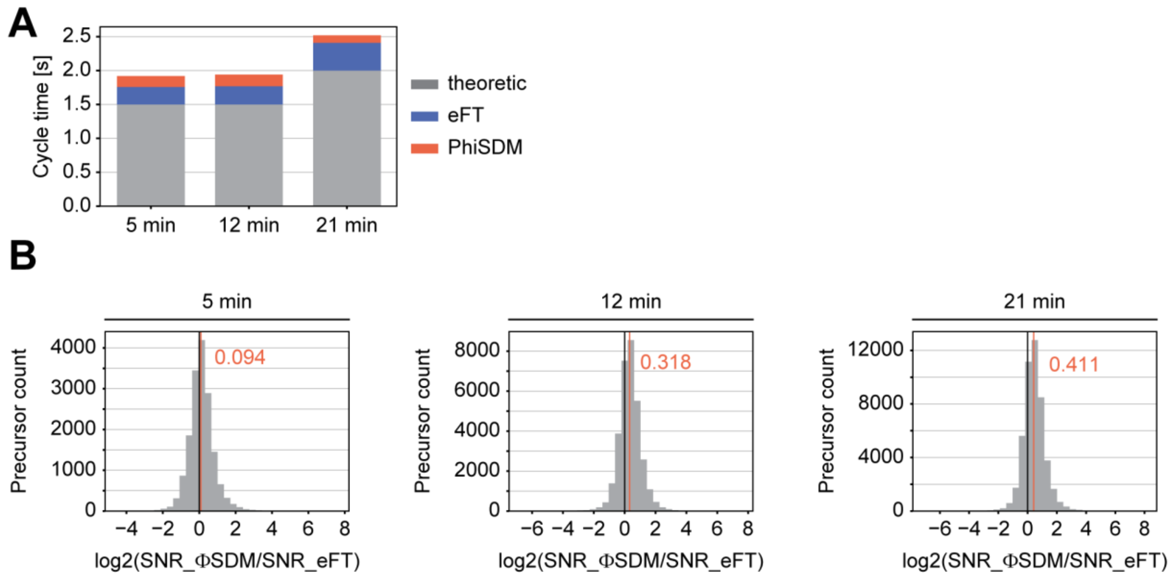

Supplementary Figure 7. Extended analysis of ΦSDM for rapid DIA proteomics. **(A)** Comparison of theoretical cycle time (gray) to the experimental cycle times for eFT (blue) and ΦSDM (orange) across the three gradient lengths. **(B)** Histogram depicting the precursor distribution across the log2 ratio of ΦSDM to eFT SNRs. Vertical zero-line depicted in black and median ΦSDM to eFT ratio indicated in red. Histograms (left to right) depict the precursor distribution for gradient lengths 5 min, 12 min and 21 min.

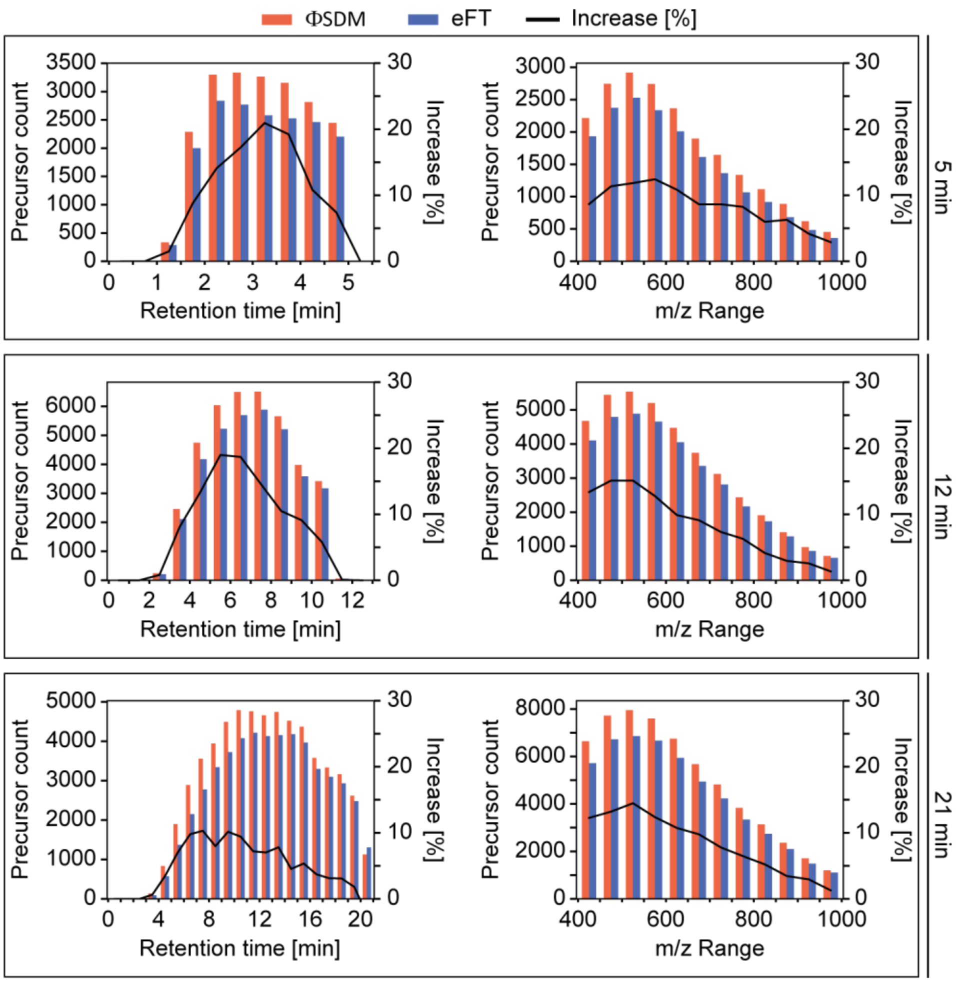

Supplementary Figure 8. Influence of ΦSDM on Identification across the *m/z* and RT dimension. Panels from top to bottom represent the precursor identifications for gradient lengths 5 min, 12 min and 21 min. Left bar charts represent precursor identification for eFT (blue) and ΦSDM (orange) along the retention time dimension with a bin size of 10 minutes. Left bar charts represent precursor identification for eFT (blue) and ΦSDM (orange) along the retention mass-to-charge (m/z) range with a bin size of 50 [m/z]. Increase in identification for ΦSDM in comparison to standard eFT is indicated in black.

Supplementary Tables

Supplementary Table 1. Description of peak neighbor pairs across *m/z* bins

m/z	100-200	200-300	300-400	400-500	500-600	600-700	700-800	800-900	900-1000	1000-1100	1100-1200	1200-1300	1300-1400	1400-1500	1500-1600	1600-1700
count	255	2051	1204	1580	3226	7779	15635	25515	37014	3853	2542	991	429	131	36	6
mean	39521.14	30222.65	24915.37	21818.48	19860.09	18377.05	17194.51	16259.38	15637.5	16294.64	15916.47	15271.59	14970	14262.58	13493.3	13806.4
std	5068.153	3674.037	2469.882	1933.02	1816.189	1794.749	1820.522	1749.219	1766.584	2406.79	2454.493	2269.96	2255.162	2017.607	1671.046	2068.913
min	30336.79	24604.63	21270.78	19022.19	17378.19	16069.28	15020.21	14168.75	13432.78	12824.84	12314.37	11837.17	11434.67	11123.46	10911.51	10651.57
25%	36078.91	27696.61	23263.41	20447.67	18573.56	17111.43	15961.92	15057.57	14364.75	14302.47	13911.77	13465.11	13185.91	12876.2	11939.56	12531.92
50%	39100.64	29484.5	24342.02	21372.46	19399.19	17869.38	16629.43	15709.5	15130.83	15800.9	15407.74	14903.52	14594.81	13824.88	13546.82	14544.63
75%	41378.28	31871.11	25923.73	22713.06	20697.59	19158.56	17853.64	16889.94	16374.31	17722.58	17470.17	16606.9	16563.1	15499.84	14869.29	14969.38
max	62361.99	48069.89	41112.73	32245.57	29858.68	29663.75	34849.01	30389.54	36222.03	26794.2	28685.95	22084.21	22364.47	20065.76	16619.92	16125.59

Supplementary Table 2. Summary statistics for calibrated mass error of fragments and precursors identified in eFT and ΦSDM

	eFT_Fragments	eFT_shared Fragments	ΦSDM_Fragments	ΦSDM_shared Fragments	eFT_Precursor	eFT_shared Precursor	ΦSDM_Precursor	ΦSDM_shared Precursor
count	305846	243149	363371	243149	59988	54709	71178	54709
mean	-0.49	-0.47	-0.18	-0.20	-0.05	-0.04	-0.08	-0.08
std	3.78	3.44	2.22	1.81	1.47	1.43	1.19	1.02
min	-16.01	-16.01	-14.37	-11.87	-7.36	-7.35	-8.36	-8.36
25%	-2.21	-1.93	-1.30	-1.09	-0.75	-0.72	-0.61	-0.55
50%	-0.09	-0.04	-0.12	-0.09	-0.03	-0.03	-0.06	-0.05
75%	1.24	1.13	0.85	0.70	0.66	0.64	0.45	0.40
max	16.28	16.09	14.15	13.45	7.76	7.74	8.28	8.28
